# Barriers to Oncofertility Care among Female Adolescent Cancer Patients in Canada

**DOI:** 10.3390/curroncol29030133

**Published:** 2022-03-03

**Authors:** Tali Sara Glazer, Fiona Schulte

**Affiliations:** 1 Cumming School of Medicine, University of Calgary, Calgary, AB T2N 4N1, Canada; fsmschul@ucalgary.ca; 2 Alberta Children’s Hospital, Calgary, AB T3B 6A8, Canada

**Keywords:** oncofertility, fertility preservation, oocyte cryopreservation, adolescent oncology

## Abstract

High survival rates in adolescent cancer patients have shifted the medical focus to the long-term outcomes of cancer treatments. Surgery, chemotherapy, and radiation increase the risk of infertility and infertility-related distress in adolescent cancer patients and survivors. The aims of this narrative review were to (1) describe the psychosocial impacts of cancer-related infertility in adolescents, (2) identify multilevel barriers to fertility preservation (FP) conversations and referrals, and (3) conclude with evidence-based clinical solutions for improving the oncofertility support available to Canadian adolescents. The results of this review revealed that FP decisions occur within the patient, parent, and health care provider (HCP) triad, and are influenced by factors such as parent attitudes, patient maturity, and HCP knowledge. Decision tools and HCP education can promote the occurrence of developmentally appropriate fertility discussions. At the systems level, cost and resource barriers prevent patients from receiving sufficient fertility information and referrals. Clinical models of care (MOCs) can define interdisciplinary roles and referral pathways to improve the integration of oncofertility services into adolescent cancer care. The continued integration of oncofertility care will ensure that all Canadian adolescents receive the exemplary medical and psychological support necessary to make empowered decisions about their own fertility.

## 1. Introduction

Oncofertility is an emerging discipline that is increasingly recognized as an essential component of adolescent cancer care [1]. The National Cancer Institute defines adolescence as patients 15–19 years of age [2]. High survival rates among adolescent cancer patients have shifted the medical focus to the long-term outcomes of cancer treatments [1]. For example, surgery, chemotherapy, and radiation increase the risk of infertility for adolescents in the years and decades following treatment [3]. Advances in oocyte cryopreservation techniques have created a viable fertility preservation (FP) option for post-pubertal female adolescents. Since 2006, the national and international cancer guidelines have advised that early fertility conversations should occur between health care providers (HCPs) and all cancer patients, and referrals to a fertility specialist should be offered to all interested patients [4]. Unfortunately, recent Canadian research [5,6,7] has shown that the majority of cancer patients do not receive the recommended standard of oncofertility care—a result of a combination of parent, patient, and health care system barriers to developmentally appropriate fertility care. The aims of this narrative review are to (1) describe the psychosocial impacts of cancer-related infertility in adolescents, (2) identify multilevel barriers to FP conversations and referrals, and (3) conclude with evidence-based clinical solutions for improving the oncofertility support available to Canadian adolescents.

## 2. Background

### 2.1. Cancer Treatment and Infertility Risk

Female adolescent and young adult (AYA) cancer survivors have significantly higher infertility rates compared to the general Canadian population [8]. Cancer treatments such as surgery, hormone therapy, chemotherapy, and radiation are associated with infertility and early menopause in adolescent girls [3]. The surgical removal of the uterus or ovaries to treat gynecologic cancers eliminates the reproductive organs required for fertility. Chemotherapy and radiation disrupt sex hormone regulation [9,10] and damage the primordial follicles [11,12,13,14,15]. Females are born with a relatively fixed number of primordial follicles that mature into oocytes during the menstrual cycle [16]. The number of primordial follicles steadily declines until a woman reaches menopause. Chemotherapy and radiation are considered ‘gonadotoxic’ because they damage the DNA and primordial follicles, while accelerating the decline in the primordial follicle population [3,17]. With depleted ovarian follicle reserves, there is a greater likelihood of infertility, temporary amenorrhea (lack of menstruation), and early menopause. The treatment-related infertility risk is dependent on the patient’s history, treatment type, cancer type, and age [18]. Cancer patients typically treated with high-intensity therapies, such as advanced-stage Hodgkin’s lymphoma patients, are at higher risk for ovarian damage [19].

### 2.2. Fertility Preservation Procedures

To mitigate the fertility risks associated with cancer treatments, adolescents can choose to participate in FP procedures that maximize the likelihood of conceiving future biological children. In Canada, FP procedures are generally provided by fertility specialists (reproductive endocrinologists) at fertility health clinics. The two main FP procedures for post-pubertal women are embryo cryopreservation (EC) and oocyte cryopreservation (OC) [20]. Both methods involve hormonal stimulation and the transvaginal surgical removal of oocytes from the ovaries. The oocyte (OC) or fertilized embryo (EC) is stored at a fertility clinic for future pregnancy through assisted reproductive technologies (ART), such as in vitro fertilization. The recent advances in vitrification techniques have improved the success rate of OC freezing, thawing, and implantation [21]. OC is the preferred FP technique for post-pubertal adolescents because it avoids the immediate requirement of a sperm donor and the ethical complications associated with sperm banks for minors. The emerging FP treatments for girls include ovarian tissue cryopreservation [22], in vitro oocyte maturation [23], and new pharmacologic therapies [17].

## 3. Psychosocial Impact of Cancer-Related Infertility

Potential and actual infertility can be highly distressing for adolescents during cancer treatment. Adolescents may perceive fertility concerns to be more distressing than the cancer diagnosis itself [24]. The majority of female adolescent cancer patients desire future children, and around 50% of adolescent cancer patients experience frustration over cancer-related fertility concerns [24,25,26]. During treatment, adolescents are concerned about the effect of the cancer treatment on their fertility status, and the likelihood of their baby inheriting cancer [24,26]. Parents may worry more about the impact of cancer treatment on their child’s future relationships than the adolescents themselves [27].

Infertility has long-term impacts on the psychosocial health of adolescents diagnosed with cancer. Infertility concerns are associated with long-term grief, depressive symptoms, and lower quality of life for female cancer survivors [28,29]. Infertility concerns are dynamic, from diagnosis to adulthood, and are influenced by age, emotional maturity, romantic relationships, and normative life expectations [30]. Infertility compromises the common adolescent desire to return to ‘normal’ after cancer treatment. Womanhood is historically associated with fertility, and adolescent cancer survivors have expressed fear of the social stigma associated with infertility [30,31]. Cancer survivors may hide their fertility concerns from their romantic partners and friends to avoid potential rejection [30,32]. Perceived fertility problems are associated with a lower likelihood of marrying and a higher likelihood of divorce in childhood cancer survivors [33].

The majority of female adolescent cancer patients desire to be aware of the impacts of their cancer treatment on fertility [26,34]. Fertility preservation counselling has been found to improve the quality of life of cancer patients and help women cope with their cancer diagnosis [35]. Positive outcomes are found for all women who receive fertility counselling by a fertility specialist, regardless of whether they chose to pursue FP [36]. Fertility information and referrals are, therefore, essential to reduce the long-term psychosocial impacts of cancer-related fertility concerns.

## 4. Unmet Needs

Since 2006, the American Society of Clinical Oncology has published oncofertility guidelines that recommend that health care providers discuss infertility with all cancer patients at reproductive age, or before puberty, as early as possible [4]. HCPs should refer every patient interested or ambivalent about FP to a fertility specialist (reproductive endocrinologist) for counselling. Various Canadian specialist groups and medical associations, such as the Canadian Fertility & Andrology Society, have created similar national and provincial FP guidelines [37,38,39].

Despite these strong guidelines, many patients do not receive adequate fertility counselling or referral after cancer diagnosis [40]. In Ontario, a population study by Korkidakis et al. [5] found that only 4% of women aged 15–39 years, with recently diagnosed breast cancer, were referred for a fertility consultation between 2000 and 2017. A similar Ontario study in lymphoma patients, by Coleman et al. [6], found that only 3.4% of AYA patients received fertility referrals between 2000 and 2018. In regards to fertility conversations, a 2015 pan-Canadian study [7] found that 71% of site-lead and 44% of general breast cancer surgeons indicated that they routinely initiated fertility discussions with patients. There is limited Canadian data on AYA fertility conversation and referral rates; however, new indicators for AYA system performance are in development [41].

Adolescents often perceive communication about infertility and FP to be insufficient [24,25,26,42]. Patients who are at a younger age at diagnosis are more likely to report unmet oncofertility information needs [43]. In addition, numerous studies have identified that females are less likely to have conversations with their HCPs about FP, and are less likely to be referred to a fertility specialist [42,44,45,46]. The 2019 Canadian Framework for Care and Support of Adolescents and Young Adults with Cancer has identified that fertility communication is an area that needs further national action [47]. Multilevel parent, patient, and system barriers prevent female adolescent cancer patients from receiving adequate fertility information and referrals from HCPs and cancer centers. The next section of this review will consider the influence of these factors on adolescent access to oncofertility care.

## 5. Barriers

### 5.1. Parent Barriers

Oncofertility conversations and decisions occur within the clinician, parent, and adolescent triad [48]. Parents are an important part of the decision-making process. Parental concerns and attitudes influence the extent of FP discussions and the outcomes of fertility decisions [48,49]. After cancer diagnosis, parents commonly prioritize immediate initiation of cancer treatment over fertility considerations [25,49]. Some parents express the desire to wait to address fertility, with the attitude that ‘we will get to it when we get to it’ [27]. Parental hesitancy around discussing fertility is related to fears of overwhelming their child and exposing them to information that is not developmentally appropriate. Their views on FP are influenced by personal beliefs [50] and cultural values [51]. Certain cultures and religions place higher value on female reproduction and encourage parents to prioritize FP.

Studies have found that oncologists are receptive to parental cues, and that they consider parental attitudes when instigating or ending fertility conversations with patients [49]. Unfortunately, parental attitudes towards FP are often incongruent with adolescent patient attitudes. Quinn et al. [26] found that the majority of parents of female adolescent cancer patients underestimated their daughter’s fertility concerns, and incorrectly assumed that their daughter would be satisfied with survivorship only. Disagreements between parents and adolescents could create potential ethical dilemmas and dissuade teenagers from accessing FP services.

### 5.2. Patient Barriers 

The patient-related barriers that inform fertility decisions include cognitive maturity, fertility knowledge, topic comfortability, and language [48,49,51,52]. Strong decision-making skills are required to navigate time-sensitive and complex fertility decisions. At diagnosis, fertility considerations compete with immediate concerns related to cancer treatment and survival [27]. Decisions are based on uncertainty, as there is no guarantee that oocyte cryopreservation will result in a successful future pregnancy. Adolescents may be less competent decision makers because their prefrontal cortex, which is the primary brain region involved in decision making, is not fully developed [53]. HCPs should consider the cognitive maturity of cancer patients during fertility discussions, to ensure that they are offering accessible and developmentally appropriate fertility information.

Fertility discussions between adolescents and HCPs are influenced by the patient’s health literacy and subject matter comfortability [48,49]. Adolescent baseline sexual and reproductive health knowledge is influenced by age and life experience. Many adolescent patients do not receive developmentally appropriate information from HCPs and institutions during their cancer experience [54]. Institutions also lack fertility resources and information in other languages [52]. HCPs are an important source of fertility information, but many adolescents are uncomfortable discussing their sexuality with clinicians, especially in the presence of their parents [48]. HCPs are receptive to the comfort levels of the patients, and may end fertility conversations prematurely if patients are embarrassed [49]. Although the guidelines recommend that all AYA cancer patients have fertility conversations with their HCPs, patient-related factors, such as limited health knowledge and embarrassment, can negatively influence the occurrence and length of fertility discussions at cancer appointments.

### 5.3. Health System Barriers

Insufficient HCP knowledge and inadequate institutional guidelines inhibit the ability of adolescents to receive adequate support for cancer-related fertility concerns [48,49,55]. HCPs identify that a lack of knowledge on FP technology and international oncofertility guidelines is a barrier to instigating fertility conversations with patients. Role confusion over which HCPs (surgeons, oncologists, or nurses) are responsible for fertility referrals is another barrier to oncofertility support [7]. In addition, many oncologists report having little knowledge of fertility clinics or specialists for patient referrals [55]. If HCPs are unaware of the fertility services in their city, they are unable to refer cancer patients to the proper support services. Appropriate fertility services may not be available for adolescents or LGBTQ2S+ patients [56].

The majority of pediatric health care providers desire standardized FP guidelines at their institutions [57]. Institutions can create clinical models of care (MOCs) to define institutional guidelines for fertility services, informational resources, and referrals [58,59]. Unfortunately, many cancer centers do not have institutional MOCs for fertility preservation [60]. The absence of official institutional guidelines likely contributes to the low HCP compliance with national and local oncofertility guidelines. In addition, many cancer centers do not have standardized referral programs or pathways to fertility specialists [18]. Fertility referral pathways are already complicated for adolescent cancer patients because teenagers fall between the medical and psychosocial boundaries of childhood and adulthood [48]. Adolescents are usually treated at pediatric cancer hospitals, while fertility specialists are available at adult centers. Standardized referral processes could ensure that there are proper networks for adolescents to find an appropriate fertility counsellor or fertility clinic. Insufficient referral guidelines have a larger effect on rural patients, who experience additional barriers to accessing fertility services [51].

The high cost of FP is a widespread system-level barrier to service access [61]. FP is expensive, and there are high costs associated with oocyte extraction, medications, oocyte storage, and future use of the eggs. Female FP is significantly more expensive than male procedures. FP coverage varies widely between Canadian provinces, and some provinces, such as British Columbia, Alberta, and Saskatchewan, offer no coverage at all [62]. Even Ontario, which arguably has the most comprehensive FP coverage program in Canada, does not cover all costs associated with FP, such as medications and oocyte storage [63]. Most adolescents have not entered the full-time workforce, and may not have the economic means to pay for FP [1]. In addition, FP concerns can occur at a time when patients and families are already under financial stress. Although Canada has publicly funded provincial health care, cancer is expensive, with hidden costs of transportation, parking, and lost wages [64]. The high costs of FP create socioeconomic disparities in accessing fertility services. In summary, the interactions between multilevel barriers and oncofertility care prevent adolescent patients from receiving the recommended cancer care outlined in the national and local oncofertility guidelines. The final section of this review will explore patient, HCP, and institutional interventions that should be implemented to improve oncofertility access for adolescents with cancer.

## 6. Recommendations for Clinical Practice

### 6.1. Decision Aids

Evidence-based decision aids (DA) can provide valuable assistance to patients navigating the complex factors involved in FP decisions [59]. Fertility decisions must be made quickly, in a period of high emotional distress and vulnerability. DAs are considered the gold standard in complicated health decisions [65]. Fertility DAs provide patients and parents with information and stepwise frameworks, and are available in online [66,67,68] or paper [69,70] formats. DAs often utilize value clarification exercises, where patients or parents evaluate which fertility outcomes are congruent with their own personal values. The goal of DAs is to help decision makers understand their priorities, reduce the decision time, improve HCP communication, and reduce long-term regret.

A decision aid entitled ‘Cancer, Fertility & Me’ was recently developed for adolescents and young females with cancer [71], and is currently undergoing validation [72]. There is also an evidence-based oncofertility decision aid for the parents of children and adolescents with cancer [73]. A DA specific to adolescents would ensure that teenagers are provided with age-appropriate resources to help them decide whether to pursue FP.

### 6.2. Health Care Provider Training

Interventions that improve HCP fertility training ensure that clinicians and nurses feel prepared to discuss fertility concerns with every adolescent cancer patient. Many pediatric oncologists express a desire to learn more about FP, especially FP options for females [49]. Education for pediatric oncologists should focus on current FP technology and sensitivity training to improve oncofertility patient communication. There are numerous new FP technologies in development, and HCPs must stay up to date with FP procedures and ART to ensure that patients are provided with all the available options. Successful FP education for HCPs should be both formal and informal, and ongoing [59]. FP training for HCPs can occur through online education programs [74,75], grand rounds [76], or improved residency training [77]. FP education is associated with more patient–provider fertility conversations and increased referrals to fertility specialists.

ENRICH is an example of a web-based oncofertility training program for HCPs that improved oncofertility care for patients [75]. The 8-week training program was developed for oncology nurses, and included lecture modules, case studies, and interactive discussions. ENRICH was successful at promoting FP conversations, referrals, and institutional change, and has since been expanded for delivery to other allied health professionals [74].

Oncofertility education should be specifically developed for clinicians and nurses that work with adolescent populations. HCPs need to understand the complex factors involved in adolescent oncofertility decision making. HCPs should know how to evaluate their patient’s health literacy, understanding of fertility, decision-making competencies, and desire for parental involvement [53]. These evaluations are important in order for HCPs to tailor their information and language to have developmentally appropriate fertility conversations. Researchers could develop standardized assessment tools or frameworks to help HCPs understand their patient’s oncofertility decision-making needs. Decision trees could also be useful to guide HCPs through the different FP options for patients [78]. Table 1 provides a summary of the oncofertility decision tools available to patients, parents, and HCPs.

### 6.3. Interdisciplinary Collaboration

Providing high-quality support to adolescents with cancer-related fertility concerns requires the interdisciplinary collaboration of pediatric oncologists, medical oncologists, fertility specialists, psychologists, and other health care providers. Psychologists are a valuable component of the interdisciplinary oncofertility team. Young women have expressed that emotional support for fertility concerns is important at all stages of cancer diagnosis, treatment, and recovery [79]. Psychologists can provide adolescents with assistance during stressful and time-constrained FP decisions [80,81]. Psychologist consultations improve decision making by helping patients understand the interplay between technical information and personal emotions during oncofertility decisions. Psychologists can also provide patients with coping skills, such as meditation, to reduce fertility-related distress. A randomized controlled trial is currently in progress to better understand the role of psychological support during oncofertility counselling [80].

The interdisciplinary integration of FP in cancer care ensures that adolescents receive fertility counselling as early as possible [18]. FP protocols can be integrated into staging and toxicity procedures, and patients can potentially complete oocyte extraction under the same general anesthetic as other procedures. Fertility conversations should also be integrated into follow-up cancer care to meet the needs of adolescent cancer survivors [59]. Increased collaboration between pediatric oncologists and fertility specialists would enhance the referral networks for adolescent cancer patients. Interdisciplinary collaboration and referral networks can be defined and supported through institutional clinical models of care.

### 6.4. Clinical Models of Care

**Figure 1:**
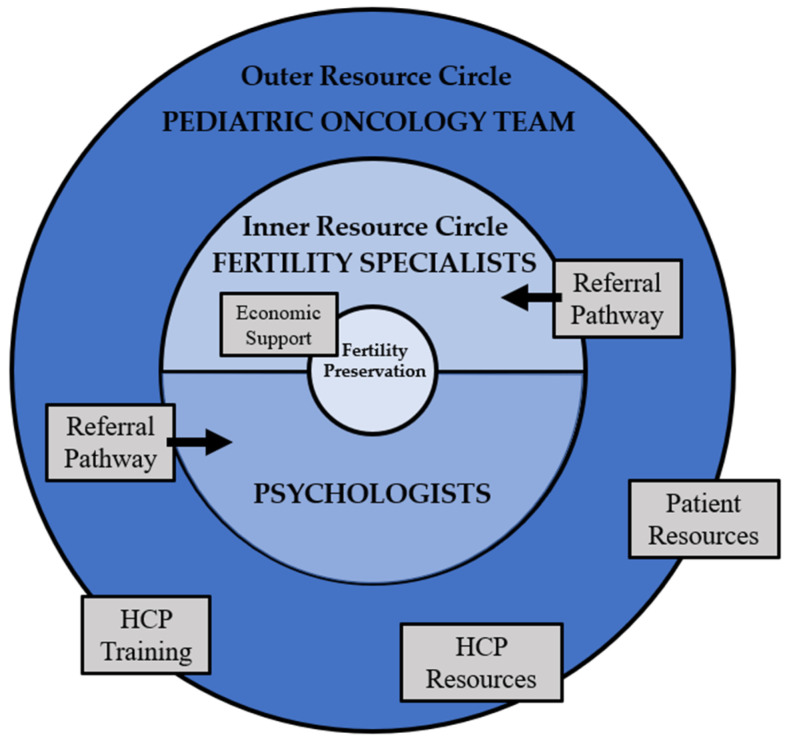
Institutional model of care framework for adolescent fertility preservation—resource circles adapted from Ronn & Holzer [86]. (Health care provider abbreviated as HPC).

Clinical MOCs and referral systems help cancer centers meet the national guidelines on FP [58,59]. MOCs define the responsibilities of different HCPs, such as surgeons, oncologists, and nurses, in providing fertility information and referrals [82]. The institutional guidelines also encourage the standard provision of fertility resources for patients through informational brochures or DAs [83]. In addition, institutional MOCs outline the recommended referral systems for fertility counselling and fertility clinics. Fertility referral systems with electronic prompts, brochures, or dedicated fertility navigators have been found to be successful at increasing referral rates [76,83]. The Duke Cancer Institute in North Carolina recently experienced a significant increase in fertility referrals after the implementation of new institutional fertility guidelines and referral pathways [84]. Cancer centers can form partnerships with fertility clinics to simplify the referral process and ensure that age-appropriate care is offered to adolescents [83]. Partnerships between cancer centers, fertility clinics, and national cancer charities can also establish price reductions for cancer patients desiring FP [76]. For example, the Canadian charity Fertile Future has partnered with the PCRM fertility clinic in Edmonton to reduce the costs of FP for women undergoing cancer treatment [85]. In addition, improved provincial coverage of FP would reduce the economic barriers for adolescent patients with cancer. In summary, clinical MOCs improve adolescent FP access through supporting the pediatric oncology team (outer resource circle) with institutional training, assessment tools, and mandated patient resources, as well as supporting psychologists and fertility specialists (inner resource circle) with defined referral pathways and funding (Figure 1).

National cancer associations can help coordinate standardized patient resources, HCP resources, and referral pathways between Canadian cancer centers [86]. In the United States, the Oncofertility Consortium and National Physicians Cooperative support translational FP research, run a national referral network, and provide resources for institutions to establish their own clinical MOCs [87]. The Canadian Oncofertility Consortium has been working to develop similar collaboration networks in Canada [88].

## 7. Conclusions

In conclusion, cancer-related infertility is detrimental to the immediate and long-term psychosocial health of many female adolescent cancer patients. Fertility-related distress can be mitigated through FP conversations between parents, patients, and HCPs, and referral consultations at fertility clinics. Although these recommendations are outlined in the national guidelines, many adolescents do not receive sufficient oncofertility support in Canada. This review has identified multilevel barriers and corresponding solutions to oncofertility access for adolescent cancer patients. Fertility preservation decisions are influenced by factors such as parent attitudes, patient maturity, and HCP knowledge. Decision tools and HCP education can promote the occurrence of developmentally appropriate fertility discussions. At the systems level, cost and resource barriers prevent patients from receiving sufficient fertility information and referrals. Clinical models of care can define interdisciplinary roles and referral pathways to improve the integration of oncofertility services into adolescent cancer care. The continued integration of oncofertility care will ensure that all Canadian adolescents receive the exemplary medical and psychological support necessary to make empowered decisions about their own fertility.

## Figures and Tables

**Table 1:** Overview of oncofertility decision tools available to patients, parents, and health care providers.

	Decision Aid	Assessment Tool	Decision Tree
Intended Use	Patients and parents	Health care providers	Health care providers
Developmental Stage Example	Multiple decision aids developed, and some validated [66,67,69] *Rank the following* *statement from 1 (not* *important) to 4* *(very important):* having my own biological child after my cancer treatment is over. Adapted from [72]	No assessment tools developed, suggested by [53] *Rank the following statement from 1 (low) to 4 (high):* my patient’s understanding of female reproduction. Adapted from [53]	One decision tree developed, but none validated [78] 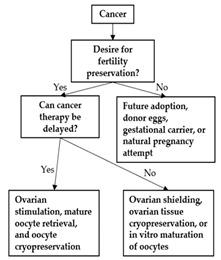 Adapted from [78]

## References

[1] Canadian Partnership Against Cancer (2017). Adolescents & Young Adults with Cancer April 2017. https://www.partnershipagainstcancer.ca/topics/adolescents-young-adults-with-cancer/.

[2] Cancer in Children and Adolescents—National Cancer Institute (2021). https://www.cancer.gov/types/childhood-cancers/child-adolescent-cancers-fact-sheet.

[3] Letourneau J., Chan S.W., Rosen M.P. (2013). Accelerating Ovarian Age: Cancer Treatment in the Premenopausal Woman. Semin. Reprod. Med..

[4] Oktay K., Harvey B., Partridge A.H., Quinn G., Reinecke J., Taylor H.S., Wallace W.H., Wang E.T., Loren A.W. (2018). Fertility Preservation in Patients with Cancer: ASCO Clinical Practice Guideline Update. J. Clin. Oncol..

[5] Korkidakis A., Lajkosz K., Green M., Strobino D., Velez M.P. (2019). Patterns of Referral for Fertility Preservation Among Female Adolescents and Young Adults with Breast Cancer: A Population-Based Study. J. Adolesc. Young-Adult Oncol..

[6] Coleman C.E., Pudwell J., McClintock C., Korkidakis A., Green M., Velez M.P. (2021). Modest Increase in Fertility Consultations in Female Adolescents and Young Adults with Lymphoma: A Population-Based Study. J. Adolesc. Young-Adult Oncol..

[7] Warner E., Yee S., Kennedy E., Glass K., Foong S., Seminsky M., Quan M.L. (2016). Oncofertility Knowledge, Attitudes, and Practices of Canadian Breast Surgeons. Ann. Surg. Oncol..

[8] Velez M.P., Richardson H., Baxter N.N., McClintock C., Greenblatt E., Barr R., Green M. (2021). Risk of infertility in female adolescents and young adults with cancer: A population-based cohort study. Hum. Reprod..

[9] Rappaport R., Brauner R., Czernichow P., Thibaud E., Renier D., Zucker J.M., Lemerle J. (1982). Effect of Hypothalamic and Pituitary Irradiation on Pubertal Development in Children with Cranial Tumors. J. Clin. Endocrinol. Metab..

[10] Constine L.S., Woolf P.D., Cann D., Mick G., McCormick K., Raubertas R.F., Rubin P. (1993). Hypothalamic-Pituitary Dysfunction after Radiation for Brain Tumors. N. Engl. J. Med..

[11] Tsai-Turton M., Luong B.T., Tan Y., Luderer U. (2007). Cyclophosphamide-Induced Apoptosis in COV434 Human Granulosa Cells Involves Oxidative Stress and Glutathione Depletion. Toxicol. Sci..

[12] Ganesan S., Keating A.F. (2016). The ovarian DNA damage repair response is induced prior to phosphoramide mustard-induced follicle depletion, and ataxia telangiectasia mutated inhibition prevents PM-induced follicle depletion. Toxicol. Appl. Pharmacol..

[13] Petrillo S.K., Desmeules P., Truong T.-Q., Devine P.J. (2011). Detection of DNA damage in oocytes of small ovarian follicles following phosphoramide mustard exposures of cultured rodent ovaries in vitro. Toxicol. Appl. Pharmacol..

[14] Wallace W.H.B., Thomson A.B., Kelsey T. (2003). The radiosensitivity of the human oocyte. Hum. Reprod..

[15] Yang W., Ma Y., Jin J., Ren P., Zhou H., Xu S., Zhang Y., Hu Z., Rong Y., Dai Y. (2021). Cyclophosphamide Exposure Causes Long-Term Detrimental Effect of Oocytes Developmental Competence Through Affecting the Epigenetic Modification and Maternal Factors’ Transcription During Oocyte Growth. Front. Cell Dev. Biol..

[16] Kerr J.B., Myers M., Anderson R. (2013). The dynamics of the primordial follicle reserve. Reproduction.

[17] Hao X., Anastácio A., Liu K., Rodriguez-Wallberg K.A. (2019). Ovarian Follicle Depletion Induced by Chemotherapy and the Investigational Stages of Potential Fertility-Protective Treatments—A Review. Int. J. Mol. Sci..

[18] Anazodo A., Ataman-Millhouse L., Jayasinghe Y., Woodruff T.K. (2018). Oncofertility-An emerging discipline rather than a special consideration. Pediatr. Blood Cancer.

[19] Behringer K., Breuer K., Reineke T., May M., Nogova L., Klimm B., Schmitz T., Wildt L., Diehl V., Engert A. (2005). Secondary Amenorrhea After Hodgkin’s Lymphoma Is Influenced by Age at Treatment, Stage of Disease, Chemotherapy Regimen, and the Use of Oral Contraceptives During Therapy: A Report from the German Hodgkin’s Lymphoma Study Group. J. Clin. Oncol..

[20] McClam M., Xiao S. (2022). Preserving Oocytes in Oncofertility. Biol. Reprod..

[21] Rienzi L., Gracia C., Maggiulli R., LaBarbera A.R., Kaser D.J., Ubaldi F.M., Vanderpoel S., Racowsky C. (2016). Oocyte, embryo and blastocyst cryopreservation in ART: Systematic review and meta-analysis comparing slow-freezing versus vitrification to produce evidence for the development of global guidance. Hum. Reprod. Update.

[22] Bahroudi Z., Zarnaghi M.R., Izadpanah M., Abedelahi A., Niknafs B., Nasrabadi H.T., Seghinsara A.M. (2021). Review of ovarian tissue cryopreservation techniques for fertility preservation. J. Gynecol. Obstet. Hum. Reprod..

[23] De Vos M., Grynberg M., Ho T.M., Yuan Y., Albertini D.F., Gilchrist R.B. (2021). Perspectives on the development and future of oocyte IVM in clinical practice. J. Assist. Reprod. Genet..

[24] Ba B.E.O., Goodwin T., Kiernan M., Hudson M.M., Dahl G.V. (2007). Concerns about infertility risks among pediatric oncology patients and their parents. Pediatr. Blood Cancer.

[25] Ellis S.J., Wakefield C.E., McLoone J.K., Robertson E.G., Cohn R.J. (2016). Fertility concerns among child and adolescent cancer survivors and their parents: A qualitative analysis. J. Psychosoc. Oncol..

[26] Quinn G.P., Knapp C., Murphy D., Sawczyn K., Sender L. (2012). Congruence of Reproductive Concerns Among Adolescents with Cancer and Parents: Pilot Testing an Adapted Instrument. Pediatrics.

[27] Stinson J.N., Jibb L., Greenberg M., Barrera M., Luca S., White M.E., Gupta A. (2015). A Qualitative Study of the Impact of Cancer on Romantic Relationships, Sexual Relationships, and Fertility: Perspectives of Canadian Adolescents and Parents During and After Treatment. J. Adolesc. Young-Adult Oncol..

[28] Gorman J.R., Su H.I., Mph S.C.R., Dominick S.A., Malcarne V. (2014). Experiencing reproductive concerns as a female cancer survivor is associated with depression. Cancer.

[29] Wenzel L., Dogan-Ates A., Habbal R., Berkowitz R., Goldstein D.P., Bernstein M., Kluhsman B.C., Osann K., Newlands E., Seckl M.J. (2005). Defining and measuring reproductive concerns of female cancer survivors. J. Natl. Cancer Inst. Monogr..

[30] Crawshaw M., Sloper P. (2010). ‘Swimming against the tide’—The influence of fertility matters on the transition to adulthood or survivorship following adolescent cancer. Eur. J. Cancer Care.

[31] Petropanagos A., Campo-Engelstein L. (2015). Tough Talk: Discussing Fertility Preservation with Adolescents and Young Adults with Cancer. J. Adolesc. Young-Adult Oncol..

[32] Zebrack B.J., Casillas J., Nohr L., Adams H., Zeltzer L.K. (2004). Fertility issues for young adult survivors of childhood cancer. Psycho-Oncology.

[33] Janson C., Leisenring W., Cox C., Termuhlen A.M., Mertens A.C., Whitton J.A., Goodman P., Zeltzer L., Robison L.L., Krull K.R. (2009). Predictors of Marriage and Divorce in Adult Survivors of Childhood Cancers: A Report from the Childhood Cancer Survivor Study. Cancer Epidemiol. Biomark. Prev..

[34] Crawshaw M., Glaser A., Hale J., Sloper P. (2009). Male and female experiences of having fertility matters raised alongside a cancer diagnosis during the teenage and young adult years. Eur. J. Cancer Care.

[35] Deshpande N.A., Braun I.M., Meyer F.L. (2015). Impact of fertility preservation counseling and treatment on psychological outcomes among women with cancer: A systematic review. Cancer.

[36] Yee S., Abrol K., McDonald M., Tonelli M., Liu K.E. (2012). Addressing Oncofertility Needs: Views of Female Cancer Patients in Fertility Preservation. J. Psychosoc. Oncol..

[37] McKillop S., Henning J., Prud’homme N., Schulte F., Labelle L., Turner J. (2020). Adolescent and Young Adult (AYA) Cancer: Clinical Practice Guideline SUPP-020.

[38] Roberts J., Ronn R., Tallon N., Holzer H.E.G. (2015). Fertility Preservation in Reproductive-Age Women Facing Gonadotoxic Treatments. Curr. Oncol..

[39] Pediatric Oncology Group of Ontario (2019). Guideline for Fertility Preservation for Patients with Cancer. https://www.pogo.ca/healthcare/practiceguidelines/fertility-preservation.

[40] Logan S., Perz J., Ussher J., Peate M., Anazodo A. (2017). A systematic review of patient oncofertility support needs in reproductive cancer patients aged 14 to 45 years of age. Psycho-Oncology.

[41] Rae C., Pole J., Gupta S., Digout C., Szwajcer D., Flanders A., Srikanthan A., Hammond C., Schacter B., Barr R.D. (2019). Development of System Performance Indicators for Adolescent and Young Adult Cancer Care and Control in Canada. Value Health.

[42] Wright C., Coad J., Morgan S., Stark D., Cable M. (2013). ‘Just in case’: The fertility information needs of teenagers and young adults with cancer. Eur. J. Cancer Care.

[43] Zebrack B. (2008). Information and service needs for young adult cancer survivors. Support. Care Cancer.

[44] Anderson R.A., Weddell A., Spoudeas H.A., Douglas C., Shalet S.M., Levitt G., Wallace W.H.B. (2008). Do doctors discuss fertility issues before they treat young patients with cancer?. Hum. Reprod..

[45] Peddie V., Porter M., Barbour R., Culligan D., Macdonald G., King D., Horn J., Bhattacharya S. (2012). Factors affecting decision making about fertility preservation after cancer diagnosis: A qualitative study. BJOG Int. J. Obstet. Gynaecol..

[46] Skaczkowski G., White V., Thompson K., Bibby H., Coory M., Orme L.M., Conyers R., Phillips M.B., Osborn M., Harrup R. (2018). Factors influencing the provision of fertility counseling and impact on quality of life in adolescents and young adults with cancer. J. Psychosoc. Oncol..

[47] Canadian Partnership Against Cancer (2019). Canadian Framework for the Care and Support of Adolescents and Young Adults with Cancer 2019. https://www.partnershipagainstcancer.ca/topics/framework-adolescents-young-adults/.

[48] Quinn G.P., Vadaparampil S.T. (2009). Fertility Preservation and Adolescent/Young Adult Cancer Patients: Physician Communication Challenges. J. Adolesc. Health.

[49] Vadaparampil S., Quinn G., King L., Wilson C., Nieder M. (2008). Barriers to fertility preservation among pediatric oncologists. Patient Educ. Couns..

[50] Li N., Jayasinghe Y., Kemertzis M.A., Moore P., Peate M. (2017). Fertility Preservation in Pediatric and Adolescent Oncology Patients: The Decision-Making Process of Parents. J. Adolesc. Young-Adult Oncol..

[51] Ussher J.M., Cummings J.R., Dryden A., Perz J. (2015). Talking about fertility in the context of cancer: Health care professional perspectives. Eur. J. Cancer Care.

[52] Murphy D., Kashal P., Quinn G., Sawczyn K., Termuhlen A. (2013). Development of a Spanish Language Fertility Educational Brochure for Pediatric Oncology Families. J. Pediatr. Adolesc. Gynecol..

[53] Quinn G.P., Murphy D., Knapp C., Stearsman D.K., Bradley-Klug K.L., Sawczyn K., Clayman M.L. (2011). Who Decides? Decision Making and Fertility Preservation in Teens with Cancer: A Review of the Literature. J. Adolesc. Health.

[54] Smith S., Davies S., Wright D., Chapman C., Mbe M.W. (2007). The experiences of teenagers and young adults with cancer—Results of 2004 conference survey. Eur. J. Oncol. Nurs..

[55] Quinn G.P., Vadaparampil S.T., Gwede C.K., Miree C., King L.M., Clayton H.B., Wilson C., Munster P. (2007). Discussion of fertility preservation with newly diagnosed patients: Oncologists’ views. J. Cancer Surviv..

[56] Tam M.W. (2021). Queering reproductive access: Reproductive justice in assisted reproductive technologies. Reprod. Health.

[57] Vadaparampil S.T., Quinn G.P., Clayton H.B., King L.M., Miree C.A. (2008). Institutional Availability of Fertility Preservation. Clin. Pediatr..

[58] Johnson R.H., Kroon L. (2013). Optimizing fertility preservation practices for adolescent and young adult cancer patients. J. Natl. Compr. Cancer Netw..

[59] Anazodo A., Laws P., Logan S., Saunders C., Travaglia J., Gerstl B., Bradford N., Cohn R., Birdsall M., Barr R. (2018). How can we improve oncofertility care for patients? A systematic scoping review of current international practice and models of care. Hum. Reprod. Update.

[60] Clayman M.L., Harper M.M., Quinn G.P., Reinecke J., Shah S. (2013). Oncofertility resources at NCI-designated comprehensive cancer centers. J. Natl. Compr. Cancer Netw..

[61] Panagiotopoulou N., Ghuman N., Sandher R., Herbert M., Stewart J. (2015). Barriers and facilitators towards fertility preservation care for cancer patients: A meta-synthesis. Eur. J. Cancer Care.

[62] Funding by Province Fertility Matters Canada (FMC). https://fertilitymatters.ca/funding-by-province.

[63] OHIP Fertility Coverage—TRIO TRIO Fertility Treatment Practice. https://triofertility.com/ohip-fertility-coverage/.

[64] Fitch M., Longo C.J. (2018). Exploring the impact of out-of-pocket costs on the quality of life of Canadian cancer patients. J. Psychosoc. Oncol..

[65] Stacey D., Légaré F., Lewis K., Barry M.J., Bennett C.L., Eden K.B., Holmes-Rovner M., Llewellyn-Thomas H., Lyddiatt A., Thomson R. (2017). Decision aids for people facing health treatment or screening decisions. Cochrane Database Syst. Rev..

[66] Garvelink M.M., Ter Kuile M.M., Louwé L.A., Hilders C.G., Stiggelbout A. (2016). Feasibility and effects of a decision aid about fertility preservation. Hum. Fertil..

[67] Ehrbar V., Germeyer A., Nawroth F., Dangel A., Findeklee S., Urech C., Rochlitz C., Stiller R., Tschudin S. (2021). Long-term effectiveness of an online decision aid for female cancer patients regarding fertility preservation: Knowledge, attitude, and decisional regret. Obstet. Gynecol. Scand..

[68] Speller B., Metcalfe K., Kennedy E.D., Facey M., Greenblatt E., Scheer A.S., Warner E., Joy A.A., Wright F.C., Baxter N.N. (2019). The “Begin Exploring Fertility Options, Risks and Expectations” (BEFORE) decision aid: Development and alpha testing of a fertility tool for premenopausal breast cancer patients. BMC Med. Inform. Decis. Mak..

[69] Peate M., Meiser B., Cheah B.C., Saunders C., Butow P., Thewes B., Hart R., Phillips K.-A., Hickey M., Friedlander M. (2012). Making hard choices easier: A prospective, multicentre study to assess the efficacy of a fertility-related decision aid in young women with early-stage breast cancer. Br. J. Cancer.

[70] Gonçalves V., Travado L., Ferreira P., Quinn G. (2019). Protocol for the development and acceptability of a fertility-related decision aid for young women with breast cancer in Portugal. BMJ Open.

[71] Making Your Fertility Preservation Decision Cancer Fertility and Me. https://cancerfertilityandme.org.uk/making-my-fertility-preservation-decisionKaGC5.

[72] Jones G.L., Hughes J., Mahmoodi N., Greenfield D., Brauten-Smith G., Skull J., Gath J., Yeomanson D., Baskind E., Snowden J.A. (2017). Observational study of the development and evaluation of a fertility preservation patient decision aid for teenage and adult women diagnosed with cancer: The Cancer, Fertility and Me research protocol. BMJ Open.

[73] Allingham C., Gillam L., McCarthy M., Zacharin M., Jayasuriya S., Heloury Y., Orme L., Sullivan M., Peate M., Jayasinghe Y. (2018). Fertility Preservation in Children and Adolescents with Cancer: Pilot of a Decision Aid for Parents of Children and Adolescents with Cancer. JMIR Pediatr. Parent..

[74] Pecoriello J., Klosky J.L., Augusto B., Santiago-Datil W., Sampson A., Reich R., Vadaparampil S., Quinn G. (2022). Evolution and growth of the ECHO (Enriching Communication skills for Health professionals in Oncofertility) program: A 5-year study in the training of oncofertility professionals. J. Cancer Surviv..

[75] Quinn G.P., Curci M.B., Reich R.R., Gwede C.K., Meade C.D., Vadaparampil S.T. (2019). The ENRICH/ECHO Working Group; ENRICH/ECHO Working Group Impact of a web-based reproductive health training program: ENRICH (Educating Nurses about Reproductive Issues in Cancer Healthcare). Psycho-Oncology.

[76] Quinn G.P., Vadaparampil S.T., Gwede C.K., Reinecke J.D., Mason T.M., Silva C. (2011). Developing a referral system for fertility preservation among patients with newly diagnosed cancer. J. Natl. Compr. Cancer Netw..

[77] Miller E.J.N., Cookingham L.M., Woodruff T.K., Ryan G.L., Summers K., Kondapalli L.A., Shah D.K. (2017). Fertility preservation training for obstetrics and gynecology fellows: A highly desired but non-standardized experience. Fertil. Res. Pract..

[78] Gardino S.L., Jeruss J.S., Woodruff T.K. (2010). Using decision trees to enhance interdisciplinary team work: The case of oncofertility. J. Assist. Reprod. Genet..

[79] Corney R.H., Swinglehurst A.J. (2013). Young childless women with breast cancer in the UK: A qualitative study of their fertility-related experiences, options, and the information given by health professionals. Psycho-Oncology.

[80] Bradford A., Woodard T.L. (2017). Novel Psychological Intervention for Decision Support in Women Considering Fertility Preservation Before Cancer Treatment. J. Adolesc. Young Adult Oncol..

[81] Razzano A., Revelli A., Piane L.D., Salvagno F., Casano S., Randaccio S., Benedetto C. (2014). Fertility preservation program before ovarotoxic oncostatic treatments: Role of the psychological support in managing emotional aspects. Gynecol. Endocrinol..

[82] Logan S., Perz J., Ussher J., Peate M., Anazodo A. (2017). Clinician provision of oncofertility support in cancer patients of a reproductive age: A systematic review. Psycho-Oncology.

[83] Reinecke J.D., Kelvin J.F., Arvey S.R., Quinn G.P., Levine J., Beck L.N., Miller A. (2012). Implementing a Systematic Approach to Meeting Patients’ Cancer and Fertility Needs: A Review of the Fertile Hope Centers of Excellence Program. J. Oncol. Pract..

[84] Dorfman C.S., Stalls J.M., Mills C., Voelkel S., Thompson M., Acharya K.S., Baker K.C., Wagner L.M., Miller N., Boswell A. (2021). Addressing Barriers to Fertility Preservation for Cancer Patients: The Role of Oncofertility Patient Navigation. J. Oncol. Navig. Surviv..

[85] Cancer Fertility Preservation|PCRM. https://edmonton.pacificfertility.ca/our-services/fertility-preservation/09ZSx.

[86] Ronn R., Holzer H.E.G. (2013). Oncofertility in Canada: An Overview of Canadian Practice and Suggested Action Plan. Curr. Oncol..

[87] Smith B.M., Duncan F.E., Ataman L., Smith K., Quinn G.P., Chang R.J., Finlayson C., Orwig K., Valli-Pulaski H., Moravek M.B. (2018). The National Physicians Cooperative: Transforming fertility management in the cancer setting and beyond. Future Oncol..

[88] Ataman L.M., Rodrigues J.K., Marinho R.M., Caetano J.P., Chehin M.B., da Motta E.L.A., Serafini P., Suzuki N., Furui T., Takae S. (2020). Creating a Global Community of Practice for Oncofertility. JCO Glob. Oncol..

